# Bis{4-[(3-phenyl­allyl­idene)amino]cyclo­hexyl}methane trichloro­methane solvate

**DOI:** 10.1107/S1600536808037380

**Published:** 2008-11-13

**Authors:** Roberto Menzel, Angela Göbel, Helmar Görls, Wolfgang Imhof

**Affiliations:** a Institute of Inorganic and Analytical Chemistry, Friedrich Schiller University, August-Bebel-Strasse 2, 07743 Jena, Germany

## Abstract

The title compound, C_31_H_38_N_2_, was prepared from bis­(4-amino­cyclo­hexyl)methane and two equivalents of cinnamaldehyde. The cyclo­hexyl groups each show a chair conformation and the α,β-unsaturated imine side chains are all-*trans* configured. Two mol­ecules of the title compound as well as two trichloromethane solvent mol­ecules are present in the asymmetric unit. The solvent mol­ecules inter­act with the diimines *via* weak C—H⋯N hydrogen bonds.

## Related literature

For general background see Imhof & Göbel (2005 ▶). For hydrogen bonding, see: Desiraju & Steiner (1999 ▶).
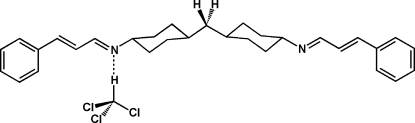


## Experimental

### 

#### Crystal data

C_31_H_38_N_2_·CHCl_3_
*M* *_r_* = 558.03
Triclinic, 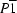

*a* = 11.7987 (6) Å
*b* = 15.7755 (7) Å
*c* = 17.1014 (6) Å
α = 80.427 (2)°
β = 85.050 (3)°
γ = 78.600 (2)°
*V* = 3072.1 (2) Å^3^
*Z* = 4
Mo *K*α radiation
μ = 0.32 mm^−1^
*T* = 183 (2) K
0.05 × 0.05 × 0.05 mm

#### Data collection

Nonius KappaCCD diffractometer
Absorption correction: none
21966 measured reflections
13946 independent reflections
7422 reflections with *I* > 2σ(*I*)
*R*_int_ = 0.042

#### Refinement

*R*[*F* ^2^ > 2σ(*F* ^2^)] = 0.087
*wR*(*F* ^2^) = 0.245
*S* = 1.02
13946 reflections
665 parameters
H-atom parameters constrained
Δρ_max_ = 1.19 e Å^−3^
Δρ_min_ = −1.10 e Å^−3^

### 

Data collection: *COLLECT* (Nonius, 1998 ▶); cell refinement: *DENZO* (Otwinowski & Minor, 1997 ▶); data reduction: *DENZO*; program(s) used to solve structure: *SHELXS97* (Sheldrick, 2008 ▶); program(s) used to refine structure: *SHELXL97* (Sheldrick, 2008 ▶); molecular graphics: *XP* (Siemens, 1990 ▶); software used to prepare material for publication: *XP*.

## Supplementary Material

Crystal structure: contains datablocks I, global. DOI: 10.1107/S1600536808037380/hg2433sup1.cif

Structure factors: contains datablocks I. DOI: 10.1107/S1600536808037380/hg2433Isup2.hkl

Additional supplementary materials:  crystallographic information; 3D view; checkCIF report

## Figures and Tables

**Table 1:** Hydrogen-bond geometry (Å, °)

*D*—H⋯*A*	*D*—H	H⋯*A*	*D*⋯*A*	*D*—H⋯*A*
C1*CA*—H1*CA*⋯N1*B*^i^	1.00	2.21	3.18 (1)	161
C1*CB*—H1*CB*⋯N1*A*^i^	1.00	2.20	3.17 (1)	165

Symmetry code: (i) 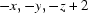.

**Fig. 1.**
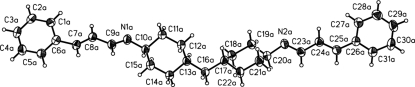
The molecular structure of the title compound showing the labelling scheme. Displacement ellipsoids are presented at the 50% probalitiy level.

## supplementary crystallographic information

### Comment

In a recent report we published the ruthenium catalyzed synthesis of chiral bis-lactams from the title compound, carbon monoxide and ethylene (Imhof & Göbel, 2005). In addition to the experimental procedure described in this reference we further purified the title compound by recrystallization from chloroform.

In the crystal structure two symmetry independent molecules of the title compound as well as two additional solvent molecules are observed in one asymmetric unit. The solvent molecules interact with the diimines *via* weak C—H···N hydrogen bonds (Desiraju & Steiner, 1999). As it is expected the cyclohexyl rings all adopt chair conformations whereas the 3-phenyl-allylidene units show an all-*trans* configuration leading to a streched shape of the molecules of the title compound (Figure 1). All bond lengths and angles are of expected values.

### Experimental

The title compund was synthesized according to a literature procedure published by some of us (Imhof & Göbel, 2005). Identity was shown by comparison of the ^1^H-NMR spectrum of the title compound with the reported spectra. Recrystallization of the title compound from anhydrous chloroform yielded the title compound as colourless crystals suitable for X-ray diffraction.

### Refinement

Hydrogen atoms were calculated in idealized positions and refined with distances of 1.00 Å (*R*_3_C—H), 0.99 Å (*R*_2_C—H_2_), and 0.95 Å (aromatic and olefinic CH). All hydrogen atoms were refined using a riding model with *U*_iso_(H) = 1.2 times *U*_iso_(C).

### Crystal data

**Table:**

C_31_H_38_N_2_·CHCl_3_	*Z* = 4
*M**_r_* = 558.03	*F*_000_ = 1184
Triclinic, *P*1	*D*_x_ = 1.206 Mg m^−^^3^
Hall symbol: -P 1	Mo *K*α radiation λ = 0.71073 Å
*a* = 11.7987 (6) Å	Cell parameters from 21966 reflections
*b* = 15.7755 (7) Å	θ = 1.8–27.5º
*c* = 17.1014 (6) Å	µ = 0.32 mm^−^^1^
α = 80.427 (2)º	*T* = 183 (2) K
β = 85.050 (3)º	Prism, colourless
γ = 78.600 (2)º	0.05 × 0.05 × 0.05 mm
*V* = 3072.1 (2) Å^3^	

### Data collection

**Table:**

Nonius KappaCCD diffractometer	7422 reflections with *I* > 2σ(*I*)
Radiation source: fine-focus sealed tube	*R*_int_ = 0.042
Monochromator: graphite	θ_max_ = 27.5º
*T* = 183(2) K	θ_min_ = 1.8º
φ and ω scans	*h* = −14→15
Absorption correction: none	*k* = −19→20
21966 measured reflections	*l* = −21→22
13946 independent reflections	

### Refinement

**Table:**

Refinement on *F*^2^	Secondary atom site location: difference Fourier map
Least-squares matrix: full	Hydrogen site location: inferred from neighbouring sites
*R*[*F*^2^ > 2σ(*F*^2^)] = 0.087	H-atom parameters constrained
*wR*(*F*^2^) = 0.245	*w* = 1/[σ^2^(*F*_o_^2^) + (0.0937*P*)^2^ + 3.8368*P*] where *P* = (*F*_o_^2^ + 2*F*_c_^2^)/3
*S* = 1.03	(Δ/σ)_max_ < 0.001
13946 reflections	Δρ_max_ = 1.19 e Å^−^^3^
665 parameters	Δρ_min_ = −1.10 e Å^−^^3^
Primary atom site location: structure-invariant direct methods	Extinction correction: none

### Special details

**Table:**

Geometry. All e.s.d.'s (except the e.s.d. in the dihedral angle between two l.s. planes) are estimated using the full covariance matrix. The cell e.s.d.'s are taken into account individually in the estimation of e.s.d.'s in distances, angles and torsion angles; correlations between e.s.d.'s in cell parameters are only used when they are defined by crystal symmetry. An approximate (isotropic) treatment of cell e.s.d.'s is used for estimating e.s.d.'s involving l.s. planes.
Refinement. Refinement of *F*^2^ against ALL reflections. The weighted *R*-factor *wR* and goodness of fit *S* are based on *F*^2^, conventional *R*-factors *R* are based on *F*, with *F* set to zero for negative *F*^2^. The threshold expression of *F*^2^ > σ(*F*^2^) is used only for calculating *R*-factors(gt) *etc*. and is not relevant to the choice of reflections for refinement. *R*-factors based on *F*^2^ are statistically about twice as large as those based on *F*, and *R*- factors based on ALL data will be even larger.

### Fractional atomic coordinates and isotropic or equivalent isotropic displacement parameters (Å^2^)

**Table:**

	*x*	*y*	*z*	*U*_iso_*/*U*_eq_	Occ. (<1)
Cl1A	−0.20422 (15)	0.01123 (10)	0.88109 (8)	0.0856 (5)	
C1CA	−0.2530 (4)	−0.0378 (3)	0.8091 (2)	0.0461 (10)	
H1CA	−0.3003	−0.0812	0.8366	0.055*	
Cl2A	−0.13634 (11)	−0.09314 (8)	0.75561 (7)	0.0647 (3)	
Cl3A	−0.34129 (12)	0.04143 (8)	0.74472 (7)	0.0674 (4)	
C1CB	0.2011 (4)	−0.0005 (3)	0.8305 (2)	0.0515 (11)	
H1CB	0.1726	−0.0572	0.8411	0.062*	0.557 (4)
H1CC	0.1800	−0.0597	0.8369	0.062*	0.443 (4)
Cl1B	0.2124 (3)	0.03085 (18)	0.92234 (15)	0.0749 (8)*	0.557 (4)
Cl2B	0.3314 (3)	−0.0183 (2)	0.7783 (2)	0.0935 (11)*	0.557 (4)
Cl3B	0.0942 (2)	0.07801 (15)	0.77647 (15)	0.0577 (7)*	0.557 (4)
Cl1	0.1498 (5)	0.0370 (3)	0.9204 (2)	0.0926 (13)*	0.443 (4)
Cl2	0.3543 (3)	0.0038 (2)	0.8121 (2)	0.0704 (11)*	0.443 (4)
Cl3	0.1358 (3)	0.0609 (2)	0.7467 (2)	0.0713 (10)*	0.443 (4)
N1A	−0.1572 (3)	0.1952 (2)	1.14817 (17)	0.0415 (8)	
N2A	−0.3270 (3)	0.3467 (2)	0.52704 (17)	0.0414 (8)	
C1A	−0.1508 (3)	0.0981 (2)	1.4641 (2)	0.0391 (9)	
H1AA	−0.2081	0.0860	1.4341	0.047*	
C2A	−0.1375 (4)	0.0566 (3)	1.5416 (2)	0.0442 (9)	
H2AA	−0.1853	0.0160	1.5643	0.053*	
C3A	−0.0550 (4)	0.0741 (2)	1.5858 (2)	0.0442 (10)	
H3AA	−0.0464	0.0455	1.6390	0.053*	
C4A	0.0149 (4)	0.1326 (3)	1.5534 (2)	0.0448 (10)	
H4AA	0.0724	0.1441	1.5838	0.054*	
C5A	0.0006 (4)	0.1748 (3)	1.4760 (2)	0.0430 (9)	
H5AA	0.0475	0.2164	1.4542	0.052*	
C6A	−0.0808 (3)	0.1577 (2)	1.4297 (2)	0.0358 (8)	
C7A	−0.0902 (3)	0.2015 (2)	1.3472 (2)	0.0379 (8)	
H7AA	−0.0585	0.2533	1.3343	0.045*	
C8A	−0.1375 (3)	0.1781 (2)	1.2883 (2)	0.0387 (9)	
H8AA	−0.1762	0.1297	1.2992	0.046*	
C9A	−0.1313 (3)	0.2245 (2)	1.2078 (2)	0.0390 (9)	
H9AA	−0.1068	0.2793	1.1993	0.047*	
C10A	−0.1391 (3)	0.2465 (2)	1.06957 (19)	0.0376 (8)	
H10A	−0.1328	0.3070	1.0767	0.045*	
C11A	−0.2413 (4)	0.2523 (3)	1.0188 (2)	0.0454 (10)	
H11B	−0.3132	0.2809	1.0453	0.055*	
H11C	−0.2505	0.1926	1.0131	0.055*	
C12A	−0.2221 (4)	0.3047 (3)	0.9363 (2)	0.0453 (10)	
H12A	−0.2883	0.3061	0.9040	0.054*	
H12B	−0.2196	0.3657	0.9421	0.054*	
C13A	−0.1109 (3)	0.2661 (2)	0.8933 (2)	0.0372 (8)	
H13B	−0.1174	0.2067	0.8832	0.045*	
C14A	−0.0085 (3)	0.2565 (3)	0.9457 (2)	0.0438 (9)	
H14A	0.0624	0.2263	0.9193	0.053*	
H14B	0.0039	0.3155	0.9513	0.053*	
C15A	−0.0277 (4)	0.2051 (3)	1.0286 (2)	0.0455 (10)	
H15A	−0.0314	0.1440	1.0239	0.055*	
H15B	0.0383	0.2038	1.0611	0.055*	
C16A	−0.0882 (4)	0.3224 (3)	0.8133 (2)	0.0423 (9)	
H16B	−0.0105	0.2978	0.7913	0.051*	
H16C	−0.0854	0.3819	0.8236	0.051*	
C17A	−0.1754 (4)	0.3311 (2)	0.7494 (2)	0.0382 (9)	
H17A	−0.2552	0.3469	0.7741	0.046*	
C18A	−0.1677 (4)	0.2467 (2)	0.7156 (2)	0.0409 (9)	
H18A	−0.1846	0.2000	0.7588	0.049*	
H18B	−0.0879	0.2284	0.6934	0.049*	
C19A	−0.2534 (3)	0.2583 (2)	0.6502 (2)	0.0390 (9)	
H19A	−0.2438	0.2030	0.6281	0.047*	
H19B	−0.3334	0.2709	0.6737	0.047*	
C20A	−0.2355 (3)	0.3322 (2)	0.5832 (2)	0.0387 (8)	
H20A	−0.1581	0.3169	0.5551	0.046*	
C21A	−0.2427 (4)	0.4169 (3)	0.6173 (2)	0.0459 (10)	
H21A	−0.2259	0.4638	0.5742	0.055*	
H21B	−0.3221	0.4353	0.6400	0.055*	
C22A	−0.1564 (4)	0.4042 (3)	0.6818 (2)	0.0454 (10)	
H22C	−0.0768	0.3903	0.6579	0.054*	
H22D	−0.1639	0.4595	0.7035	0.054*	
C23A	−0.2978 (3)	0.3601 (2)	0.4536 (2)	0.0377 (8)	
H23C	−0.2181	0.3570	0.4375	0.045*	
C24A	−0.3826 (4)	0.3801 (2)	0.3935 (2)	0.0387 (8)	
H24A	−0.4621	0.3841	0.4101	0.046*	
C25A	−0.3535 (4)	0.3931 (2)	0.3157 (2)	0.0398 (9)	
H25A	−0.2731	0.3876	0.3013	0.048*	
C26A	−0.4314 (3)	0.4148 (2)	0.2500 (2)	0.0361 (8)	
C27A	−0.5512 (4)	0.4289 (3)	0.2610 (2)	0.0432 (9)	
H27B	−0.5856	0.4240	0.3135	0.052*	
C28A	−0.6215 (4)	0.4501 (3)	0.1973 (3)	0.0527 (11)	
H28A	−0.7034	0.4602	0.2061	0.063*	
C29A	−0.5722 (4)	0.4565 (3)	0.1206 (2)	0.0535 (11)	
H29A	−0.6201	0.4709	0.0766	0.064*	
C30A	−0.4547 (4)	0.4421 (3)	0.1083 (2)	0.0565 (12)	
H30B	−0.4211	0.4462	0.0556	0.068*	
C31A	−0.3840 (4)	0.4216 (3)	0.1719 (2)	0.0482 (10)	
H31A	−0.3022	0.4120	0.1623	0.058*	
N1B	0.3897 (3)	0.1944 (2)	1.13968 (17)	0.0457 (8)	
N2B	0.1718 (3)	0.3737 (2)	0.52557 (18)	0.0445 (8)	
C1B	0.3739 (4)	0.1107 (3)	1.4516 (2)	0.0440 (9)	
H1BA	0.3202	0.1016	1.4168	0.053*	
C2B	0.3636 (4)	0.0788 (3)	1.5316 (2)	0.0499 (10)	
H2BA	0.3036	0.0474	1.5514	0.060*	
C3B	0.4405 (4)	0.0923 (3)	1.5829 (2)	0.0502 (11)	
H3BA	0.4337	0.0696	1.6378	0.060*	
C4B	0.5262 (4)	0.1384 (3)	1.5547 (2)	0.0495 (11)	
H4BA	0.5781	0.1485	1.5901	0.059*	
C5B	0.5372 (4)	0.1703 (2)	1.4742 (2)	0.0413 (9)	
H5BA	0.5968	0.2023	1.4550	0.050*	
C6B	0.4619 (3)	0.1561 (2)	1.4213 (2)	0.0374 (8)	
C7B	0.4796 (3)	0.1887 (2)	1.3365 (2)	0.0403 (9)	
H7BA	0.5408	0.2203	1.3220	0.048*	
C8B	0.4188 (4)	0.1786 (3)	1.2778 (2)	0.0425 (9)	
H8BA	0.3548	0.1497	1.2909	0.051*	
C9B	0.4451 (4)	0.2094 (3)	1.1947 (2)	0.0424 (9)	
H9BA	0.5057	0.2418	1.1810	0.051*	
C10B	0.4256 (3)	0.2249 (3)	1.0578 (2)	0.0433 (9)	
H10B	0.4890	0.2587	1.0577	0.052*	
C11B	0.3236 (4)	0.2845 (3)	1.0155 (2)	0.0495 (10)	
H11A	0.2963	0.3360	1.0429	0.059*	
H11D	0.2589	0.2525	1.0176	0.059*	
C12B	0.3589 (4)	0.3152 (3)	0.9288 (2)	0.0508 (10)	
H12C	0.2905	0.3515	0.9022	0.061*	
H12D	0.4179	0.3524	0.9271	0.061*	
C13B	0.4077 (3)	0.2398 (3)	0.8834 (2)	0.0394 (9)	
H13A	0.3446	0.2067	0.8804	0.047*	
C14B	0.5055 (4)	0.1779 (3)	0.9279 (2)	0.0524 (11)	
H14C	0.5310	0.1261	0.9007	0.063*	
H14D	0.5722	0.2078	0.9262	0.063*	
C15B	0.4705 (4)	0.1475 (3)	1.0142 (2)	0.0530 (11)	
H15C	0.4094	0.1120	1.0163	0.064*	
H15D	0.5381	0.1100	1.0408	0.064*	
C16B	0.4499 (3)	0.2722 (3)	0.7983 (2)	0.0411 (9)	
H16A	0.5036	0.3125	0.8010	0.049*	
H16D	0.4951	0.2213	0.7756	0.049*	
C17B	0.3572 (3)	0.3192 (2)	0.7408 (2)	0.0370 (8)	
H17B	0.3090	0.3689	0.7650	0.044*	
C18B	0.2778 (4)	0.2588 (3)	0.7262 (2)	0.0447 (10)	
H18C	0.2339	0.2417	0.7764	0.054*	
H18D	0.3255	0.2049	0.7099	0.054*	
C19B	0.1925 (4)	0.3011 (3)	0.6625 (2)	0.0477 (10)	
H19C	0.1460	0.2582	0.6534	0.057*	
H19D	0.1390	0.3514	0.6810	0.057*	
C20B	0.2557 (3)	0.3323 (2)	0.5850 (2)	0.0398 (9)	
H20B	0.3082	0.2813	0.5655	0.048*	
C21B	0.3270 (4)	0.3984 (3)	0.5996 (2)	0.0434 (9)	
H21C	0.3688	0.4189	0.5495	0.052*	
H21D	0.2749	0.4497	0.6175	0.052*	
C22B	0.4135 (3)	0.3570 (3)	0.6623 (2)	0.0432 (9)	
H22A	0.4566	0.4016	0.6723	0.052*	
H22B	0.4700	0.3096	0.6415	0.052*	
C23B	0.1876 (4)	0.3462 (2)	0.4590 (2)	0.0416 (9)	
H23A	0.2504	0.2998	0.4515	0.050*	
C24B	0.1127 (4)	0.3837 (3)	0.3941 (2)	0.0417 (9)	
H24B	0.0478	0.4282	0.4025	0.050*	
C25B	0.1320 (3)	0.3576 (2)	0.3230 (2)	0.0407 (9)	
H25B	0.1949	0.3105	0.3178	0.049*	
C26B	0.0667 (3)	0.3937 (2)	0.2519 (2)	0.0357 (8)	
C27B	−0.0348 (3)	0.4559 (3)	0.2529 (2)	0.0424 (9)	
H27A	−0.0656	0.4748	0.3017	0.051*	
C28B	−0.0914 (4)	0.4907 (3)	0.1841 (2)	0.0487 (10)	
H28B	−0.1606	0.5332	0.1859	0.058*	
C29B	−0.0476 (4)	0.4638 (3)	0.1125 (2)	0.0468 (10)	
H29B	−0.0858	0.4886	0.0651	0.056*	
C30B	0.0510 (4)	0.4015 (3)	0.1104 (2)	0.0461 (10)	
H30A	0.0806	0.3825	0.0616	0.055*	
C31B	0.1076 (4)	0.3662 (3)	0.1794 (2)	0.0431 (9)	
H31B	0.1754	0.3224	0.1773	0.052*	

### Atomic displacement parameters (Å^2^)

**Table:**

	*U*^11^	*U*^22^	*U*^33^	*U*^12^	*U*^13^	*U*^23^
Cl1A	0.1030 (12)	0.0857 (10)	0.0740 (9)	−0.0116 (8)	−0.0355 (8)	−0.0209 (7)
C1CA	0.047 (3)	0.047 (2)	0.041 (2)	−0.0064 (19)	−0.0025 (18)	−0.0005 (17)
Cl2A	0.0523 (7)	0.0630 (7)	0.0708 (7)	−0.0030 (6)	0.0100 (6)	−0.0035 (6)
Cl3A	0.0736 (9)	0.0570 (7)	0.0629 (7)	0.0066 (6)	−0.0216 (6)	0.0021 (5)
C1CB	0.067 (3)	0.042 (2)	0.044 (2)	−0.012 (2)	−0.005 (2)	−0.0004 (18)
N1A	0.050 (2)	0.0425 (18)	0.0315 (16)	−0.0120 (16)	−0.0056 (14)	0.0001 (13)
N2A	0.045 (2)	0.0457 (18)	0.0337 (17)	−0.0096 (15)	−0.0099 (14)	−0.0024 (14)
C1A	0.039 (2)	0.046 (2)	0.0362 (19)	−0.0130 (18)	−0.0005 (16)	−0.0099 (16)
C2A	0.051 (3)	0.045 (2)	0.036 (2)	−0.0148 (19)	0.0088 (17)	−0.0038 (16)
C3A	0.062 (3)	0.040 (2)	0.0282 (18)	−0.008 (2)	−0.0022 (17)	−0.0016 (15)
C4A	0.056 (3)	0.046 (2)	0.0344 (19)	−0.012 (2)	−0.0076 (18)	−0.0061 (17)
C5A	0.051 (3)	0.042 (2)	0.039 (2)	−0.0189 (19)	−0.0055 (18)	−0.0039 (16)
C6A	0.044 (2)	0.0287 (18)	0.0340 (18)	−0.0058 (16)	0.0018 (16)	−0.0066 (14)
C7A	0.047 (2)	0.0328 (19)	0.0327 (18)	−0.0078 (17)	−0.0008 (16)	−0.0021 (15)
C8A	0.045 (2)	0.039 (2)	0.0329 (19)	−0.0109 (18)	−0.0018 (16)	−0.0037 (15)
C9A	0.043 (2)	0.039 (2)	0.0344 (19)	−0.0070 (17)	−0.0065 (16)	−0.0010 (15)
C10A	0.046 (2)	0.038 (2)	0.0287 (17)	−0.0133 (17)	−0.0053 (16)	0.0016 (15)
C11A	0.040 (2)	0.059 (3)	0.036 (2)	−0.014 (2)	−0.0059 (17)	0.0038 (18)
C12A	0.046 (2)	0.056 (2)	0.0325 (19)	−0.011 (2)	−0.0089 (17)	0.0023 (17)
C13A	0.044 (2)	0.0362 (19)	0.0328 (18)	−0.0119 (17)	−0.0062 (16)	−0.0007 (15)
C14A	0.038 (2)	0.058 (2)	0.0333 (19)	−0.0087 (19)	−0.0048 (16)	0.0002 (17)
C15A	0.046 (2)	0.053 (2)	0.036 (2)	−0.008 (2)	−0.0099 (17)	0.0004 (17)
C16A	0.048 (2)	0.050 (2)	0.0323 (19)	−0.0203 (19)	−0.0075 (17)	−0.0005 (16)
C17A	0.048 (2)	0.037 (2)	0.0302 (18)	−0.0134 (17)	−0.0068 (16)	0.0020 (15)
C18A	0.049 (2)	0.036 (2)	0.038 (2)	−0.0098 (18)	−0.0136 (17)	−0.0018 (16)
C19A	0.042 (2)	0.038 (2)	0.039 (2)	−0.0094 (17)	−0.0098 (16)	−0.0048 (16)
C20A	0.040 (2)	0.044 (2)	0.0320 (18)	−0.0095 (17)	−0.0056 (16)	−0.0041 (15)
C21A	0.058 (3)	0.040 (2)	0.040 (2)	−0.0144 (19)	−0.0134 (18)	0.0033 (16)
C22A	0.066 (3)	0.043 (2)	0.0326 (19)	−0.024 (2)	−0.0125 (18)	0.0005 (16)
C23A	0.042 (2)	0.0363 (19)	0.037 (2)	−0.0098 (17)	−0.0080 (16)	−0.0071 (15)
C24A	0.043 (2)	0.038 (2)	0.037 (2)	−0.0115 (17)	−0.0050 (16)	−0.0051 (15)
C25A	0.040 (2)	0.041 (2)	0.039 (2)	−0.0079 (17)	−0.0040 (16)	−0.0055 (16)
C26A	0.042 (2)	0.0332 (19)	0.0330 (18)	−0.0058 (16)	−0.0037 (16)	−0.0048 (14)
C27A	0.045 (2)	0.044 (2)	0.040 (2)	−0.0058 (18)	−0.0054 (17)	−0.0049 (17)
C28A	0.044 (3)	0.055 (3)	0.057 (3)	−0.006 (2)	−0.011 (2)	−0.002 (2)
C29A	0.063 (3)	0.051 (2)	0.046 (2)	−0.010 (2)	−0.023 (2)	0.0042 (19)
C30A	0.072 (3)	0.063 (3)	0.032 (2)	−0.012 (2)	−0.008 (2)	0.0024 (19)
C31A	0.045 (3)	0.058 (3)	0.040 (2)	−0.009 (2)	−0.0050 (18)	0.0000 (18)
N1B	0.050 (2)	0.057 (2)	0.0311 (16)	−0.0172 (17)	−0.0049 (14)	0.0007 (14)
N2B	0.048 (2)	0.0442 (18)	0.0397 (18)	−0.0090 (16)	−0.0117 (15)	0.0046 (14)
C1B	0.052 (3)	0.043 (2)	0.040 (2)	−0.0170 (19)	−0.0079 (18)	−0.0033 (17)
C2B	0.062 (3)	0.043 (2)	0.045 (2)	−0.018 (2)	0.002 (2)	−0.0023 (18)
C3B	0.071 (3)	0.041 (2)	0.036 (2)	−0.008 (2)	−0.007 (2)	−0.0005 (17)
C4B	0.062 (3)	0.049 (2)	0.039 (2)	−0.005 (2)	−0.018 (2)	−0.0074 (18)
C5B	0.045 (2)	0.037 (2)	0.044 (2)	−0.0094 (18)	−0.0100 (17)	−0.0049 (16)
C6B	0.046 (2)	0.0311 (18)	0.0361 (19)	−0.0081 (17)	−0.0071 (16)	−0.0041 (15)
C7B	0.045 (2)	0.037 (2)	0.039 (2)	−0.0096 (18)	−0.0088 (17)	−0.0015 (16)
C8B	0.043 (2)	0.048 (2)	0.037 (2)	−0.0125 (19)	−0.0035 (17)	−0.0033 (16)
C9B	0.047 (2)	0.045 (2)	0.036 (2)	−0.0102 (19)	−0.0055 (17)	−0.0028 (16)
C10B	0.042 (2)	0.057 (2)	0.0330 (19)	−0.0173 (19)	−0.0028 (16)	−0.0013 (17)
C11B	0.048 (3)	0.061 (3)	0.036 (2)	0.000 (2)	−0.0038 (18)	−0.0075 (18)
C12B	0.054 (3)	0.055 (3)	0.036 (2)	0.001 (2)	0.0005 (18)	−0.0026 (18)
C13B	0.037 (2)	0.049 (2)	0.0322 (18)	−0.0074 (18)	−0.0071 (15)	−0.0040 (16)
C14B	0.056 (3)	0.057 (3)	0.037 (2)	0.005 (2)	−0.0060 (19)	−0.0039 (18)
C15B	0.057 (3)	0.056 (3)	0.039 (2)	0.003 (2)	−0.0100 (19)	−0.0006 (19)
C16B	0.036 (2)	0.052 (2)	0.0333 (19)	−0.0065 (18)	−0.0044 (16)	−0.0020 (16)
C17B	0.037 (2)	0.040 (2)	0.0329 (18)	−0.0060 (17)	−0.0027 (15)	−0.0039 (15)
C18B	0.053 (3)	0.047 (2)	0.0349 (19)	−0.017 (2)	−0.0111 (17)	0.0053 (16)
C19B	0.050 (3)	0.056 (2)	0.041 (2)	−0.025 (2)	−0.0122 (18)	0.0027 (18)
C20B	0.045 (2)	0.037 (2)	0.0353 (19)	−0.0058 (17)	−0.0118 (16)	0.0021 (15)
C21B	0.044 (2)	0.044 (2)	0.040 (2)	−0.0136 (19)	−0.0086 (17)	0.0069 (17)
C22B	0.041 (2)	0.051 (2)	0.038 (2)	−0.0145 (19)	−0.0075 (17)	0.0020 (17)
C23B	0.047 (2)	0.035 (2)	0.042 (2)	−0.0130 (18)	−0.0085 (17)	0.0048 (16)
C24B	0.045 (2)	0.041 (2)	0.040 (2)	−0.0160 (18)	−0.0107 (17)	0.0049 (16)
C25B	0.043 (2)	0.035 (2)	0.044 (2)	−0.0095 (17)	−0.0127 (17)	0.0013 (16)
C26B	0.040 (2)	0.0328 (19)	0.0360 (19)	−0.0139 (17)	−0.0070 (16)	0.0002 (15)
C27B	0.044 (2)	0.046 (2)	0.039 (2)	−0.0104 (19)	−0.0036 (17)	−0.0064 (17)
C28B	0.043 (2)	0.050 (2)	0.050 (2)	−0.0014 (19)	−0.0115 (19)	−0.0032 (19)
C29B	0.051 (3)	0.051 (2)	0.040 (2)	−0.016 (2)	−0.0167 (18)	0.0055 (18)
C30B	0.043 (2)	0.059 (3)	0.037 (2)	−0.014 (2)	−0.0031 (17)	−0.0069 (18)
C31B	0.040 (2)	0.047 (2)	0.043 (2)	−0.0105 (18)	−0.0031 (17)	−0.0070 (17)

### Geometric parameters (Å, °)

**Table:**

Cl1A—C1CA	1.749 (4)	C28A—H28A	0.9500
C1CA—Cl2A	1.751 (4)	C29A—C30A	1.363 (7)
C1CA—Cl3A	1.757 (4)	C29A—H29A	0.9500
C1CA—H1CA	1.0000	C30A—C31A	1.385 (6)
C1CB—Cl2B	1.708 (6)	C30A—H30B	0.9500
C1CB—Cl3	1.738 (5)	C31A—H31A	0.9500
C1CB—Cl1B	1.747 (5)	N1B—C9B	1.269 (5)
C1CB—Cl1	1.755 (6)	N1B—C10B	1.459 (5)
C1CB—Cl3B	1.783 (5)	N2B—C23B	1.271 (5)
C1CB—Cl2	1.821 (6)	N2B—C20B	1.462 (4)
C1CB—H1CB	1.0000	C1B—C2B	1.380 (5)
C1CB—H1CC	1.0001	C1B—C6B	1.391 (5)
N1A—C9A	1.270 (5)	C1B—H1BA	0.9500
N1A—C10A	1.471 (4)	C2B—C3B	1.384 (6)
N2A—C23A	1.268 (5)	C2B—H2BA	0.9500
N2A—C20A	1.467 (4)	C3B—C4B	1.368 (6)
C1A—C2A	1.384 (5)	C3B—H3BA	0.9500
C1A—C6A	1.396 (5)	C4B—C5B	1.389 (5)
C1A—H1AA	0.9500	C4B—H4BA	0.9500
C2A—C3A	1.377 (5)	C5B—C6B	1.394 (5)
C2A—H2AA	0.9500	C5B—H5BA	0.9500
C3A—C4A	1.376 (6)	C6B—C7B	1.468 (5)
C3A—H3AA	0.9500	C7B—C8B	1.330 (5)
C4A—C5A	1.387 (5)	C7B—H7BA	0.9500
C4A—H4AA	0.9500	C8B—C9B	1.452 (5)
C5A—C6A	1.388 (5)	C8B—H8BA	0.9500
C5A—H5AA	0.9500	C9B—H9BA	0.9500
C6A—C7A	1.467 (5)	C10B—C15B	1.519 (6)
C7A—C8A	1.328 (5)	C10B—C11B	1.528 (6)
C7A—H7AA	0.9500	C10B—H10B	1.0000
C8A—C9A	1.451 (5)	C11B—C12B	1.530 (5)
C8A—H8AA	0.9500	C11B—H11A	0.9900
C9A—H9AA	0.9500	C11B—H11D	0.9900
C10A—C15A	1.516 (6)	C12B—C13B	1.519 (6)
C10A—C11A	1.526 (5)	C12B—H12C	0.9900
C10A—H10A	1.0000	C12B—H12D	0.9900
C11A—C12A	1.535 (5)	C13B—C14B	1.525 (5)
C11A—H11B	0.9900	C13B—C16B	1.535 (5)
C11A—H11C	0.9900	C13B—H13A	1.0000
C12A—C13A	1.516 (6)	C14B—C15B	1.521 (5)
C12A—H12A	0.9900	C14B—H14C	0.9900
C12A—H12B	0.9900	C14B—H14D	0.9900
C13A—C16A	1.535 (5)	C15B—H15C	0.9900
C13A—C14A	1.535 (5)	C15B—H15D	0.9900
C13A—H13B	1.0000	C16B—C17B	1.528 (5)
C14A—C15A	1.535 (5)	C16B—H16A	0.9900
C14A—H14A	0.9900	C16B—H16D	0.9900
C14A—H14B	0.9900	C17B—C18B	1.524 (5)
C15A—H15A	0.9900	C17B—C22B	1.528 (5)
C15A—H15B	0.9900	C17B—H17B	1.0000
C16A—C17A	1.536 (5)	C18B—C19B	1.525 (5)
C16A—H16B	0.9900	C18B—H18C	0.9900
C16A—H16C	0.9900	C18B—H18D	0.9900
C17A—C18A	1.520 (5)	C19B—C20B	1.520 (5)
C17A—C22A	1.527 (5)	C19B—H19C	0.9900
C17A—H17A	1.0000	C19B—H19D	0.9900
C18A—C19A	1.536 (5)	C20B—C21B	1.526 (5)
C18A—H18A	0.9900	C20B—H20B	1.0000
C18A—H18B	0.9900	C21B—C22B	1.520 (5)
C19A—C20A	1.526 (5)	C21B—H21C	0.9900
C19A—H19A	0.9900	C21B—H21D	0.9900
C19A—H19B	0.9900	C22B—H22A	0.9900
C20A—C21A	1.529 (5)	C22B—H22B	0.9900
C20A—H20A	1.0000	C23B—C24B	1.452 (5)
C21A—C22A	1.527 (5)	C23B—H23A	0.9500
C21A—H21A	0.9900	C24B—C25B	1.334 (5)
C21A—H21B	0.9900	C24B—H24B	0.9500
C22A—H22C	0.9900	C25B—C26B	1.468 (5)
C22A—H22D	0.9900	C25B—H25B	0.9500
C23A—C24A	1.450 (5)	C26B—C27B	1.391 (5)
C23A—H23C	0.9500	C26B—C31B	1.397 (5)
C24A—C25A	1.337 (5)	C27B—C28B	1.381 (5)
C24A—H24A	0.9500	C27B—H27A	0.9500
C25A—C26A	1.466 (5)	C28B—C29B	1.386 (6)
C25A—H25A	0.9500	C28B—H28B	0.9500
C26A—C27A	1.388 (5)	C29B—C30B	1.368 (6)
C26A—C31A	1.398 (5)	C29B—H29B	0.9500
C27A—C28A	1.384 (5)	C30B—C31B	1.384 (5)
C27A—H27B	0.9500	C30B—H30A	0.9500
C28A—C29A	1.384 (6)	C31B—H31B	0.9500
			
Cl1A—C1CA—Cl2A	110.9 (2)	C27A—C26A—C25A	123.3 (3)
Cl1A—C1CA—Cl3A	110.1 (2)	C31A—C26A—C25A	119.1 (4)
Cl2A—C1CA—Cl3A	110.5 (2)	C28A—C27A—C26A	121.4 (4)
Cl1A—C1CA—H1CA	108.4	C28A—C27A—H27B	119.3
Cl2A—C1CA—H1CA	108.4	C26A—C27A—H27B	119.3
Cl3A—C1CA—H1CA	108.4	C29A—C28A—C27A	119.7 (4)
Cl2B—C1CB—Cl3	90.9 (3)	C29A—C28A—H28A	120.1
Cl2B—C1CB—Cl1B	112.5 (3)	C27A—C28A—H28A	120.1
Cl3—C1CB—Cl1B	129.0 (3)	C30A—C29A—C28A	119.9 (4)
Cl2B—C1CB—Cl1	136.2 (3)	C30A—C29A—H29A	120.0
Cl3—C1CB—Cl1	115.0 (3)	C28A—C29A—H29A	120.0
Cl1B—C1CB—Cl1	24.00 (16)	C29A—C30A—C31A	120.6 (4)
Cl2B—C1CB—Cl3B	112.8 (3)	C29A—C30A—H30B	119.7
Cl3—C1CB—Cl3B	23.67 (13)	C31A—C30A—H30B	119.7
Cl1B—C1CB—Cl3B	109.3 (3)	C30A—C31A—C26A	120.8 (4)
Cl1—C1CB—Cl3B	92.1 (3)	C30A—C31A—H31A	119.6
Cl2B—C1CB—Cl2	26.34 (14)	C26A—C31A—H31A	119.6
Cl3—C1CB—Cl2	104.8 (3)	C9B—N1B—C10B	118.0 (3)
Cl1B—C1CB—Cl2	86.5 (2)	C23B—N2B—C20B	116.4 (3)
Cl1—C1CB—Cl2	109.9 (3)	C2B—C1B—C6B	120.7 (4)
Cl3B—C1CB—Cl2	120.8 (3)	C2B—C1B—H1BA	119.7
Cl2B—C1CB—H1CB	107.3	C6B—C1B—H1BA	119.7
Cl3—C1CB—H1CB	107.6	C1B—C2B—C3B	120.2 (4)
Cl1B—C1CB—H1CB	107.3	C1B—C2B—H2BA	119.9
Cl1—C1CB—H1CB	98.0	C3B—C2B—H2BA	119.9
Cl3B—C1CB—H1CB	107.3	C4B—C3B—C2B	120.1 (4)
Cl2—C1CB—H1CB	121.9	C4B—C3B—H3BA	120.0
Cl2B—C1CB—H1CC	100.9	C2B—C3B—H3BA	120.0
Cl3—C1CB—H1CC	107.1	C3B—C4B—C5B	119.9 (4)
Cl1B—C1CB—H1CC	111.5	C3B—C4B—H4BA	120.0
Cl1—C1CB—H1CC	103.8	C5B—C4B—H4BA	120.0
Cl3B—C1CB—H1CC	109.6	C4B—C5B—C6B	120.9 (4)
Cl2—C1CB—H1CC	116.4	C4B—C5B—H5BA	119.6
H1CB—C1CB—H1CC	6.5	C6B—C5B—H5BA	119.6
C9A—N1A—C10A	116.9 (3)	C1B—C6B—C5B	118.2 (3)
C23A—N2A—C20A	117.5 (3)	C1B—C6B—C7B	123.1 (3)
C2A—C1A—C6A	120.7 (3)	C5B—C6B—C7B	118.7 (3)
C2A—C1A—H1AA	119.7	C8B—C7B—C6B	126.8 (4)
C6A—C1A—H1AA	119.7	C8B—C7B—H7BA	116.6
C3A—C2A—C1A	120.1 (4)	C6B—C7B—H7BA	116.6
C3A—C2A—H2AA	119.9	C7B—C8B—C9B	123.5 (4)
C1A—C2A—H2AA	119.9	C7B—C8B—H8BA	118.2
C4A—C3A—C2A	120.4 (3)	C9B—C8B—H8BA	118.2
C4A—C3A—H3AA	119.8	N1B—C9B—C8B	122.0 (4)
C2A—C3A—H3AA	119.8	N1B—C9B—H9BA	119.0
C3A—C4A—C5A	119.3 (4)	C8B—C9B—H9BA	119.0
C3A—C4A—H4AA	120.3	N1B—C10B—C15B	110.1 (3)
C5A—C4A—H4AA	120.3	N1B—C10B—C11B	110.0 (3)
C4A—C5A—C6A	121.5 (4)	C15B—C10B—C11B	109.7 (3)
C4A—C5A—H5AA	119.2	N1B—C10B—H10B	109.0
C6A—C5A—H5AA	119.2	C15B—C10B—H10B	109.0
C5A—C6A—C1A	117.9 (3)	C11B—C10B—H10B	109.0
C5A—C6A—C7A	119.7 (3)	C10B—C11B—C12B	110.7 (4)
C1A—C6A—C7A	122.4 (3)	C10B—C11B—H11A	109.5
C8A—C7A—C6A	128.3 (3)	C12B—C11B—H11A	109.5
C8A—C7A—H7AA	115.9	C10B—C11B—H11D	109.5
C6A—C7A—H7AA	115.9	C12B—C11B—H11D	109.5
C7A—C8A—C9A	121.7 (3)	H11A—C11B—H11D	108.1
C7A—C8A—H8AA	119.2	C13B—C12B—C11B	112.8 (3)
C9A—C8A—H8AA	119.2	C13B—C12B—H12C	109.0
N1A—C9A—C8A	123.1 (3)	C11B—C12B—H12C	109.0
N1A—C9A—H9AA	118.5	C13B—C12B—H12D	109.0
C8A—C9A—H9AA	118.5	C11B—C12B—H12D	109.0
N1A—C10A—C15A	110.0 (3)	H12C—C12B—H12D	107.8
N1A—C10A—C11A	110.4 (3)	C12B—C13B—C14B	109.9 (3)
C15A—C10A—C11A	110.2 (3)	C12B—C13B—C16B	111.8 (3)
N1A—C10A—H10A	108.8	C14B—C13B—C16B	110.6 (3)
C15A—C10A—H10A	108.8	C12B—C13B—H13A	108.1
C11A—C10A—H10A	108.8	C14B—C13B—H13A	108.1
C10A—C11A—C12A	110.6 (3)	C16B—C13B—H13A	108.1
C10A—C11A—H11B	109.5	C15B—C14B—C13B	112.9 (4)
C12A—C11A—H11B	109.5	C15B—C14B—H14C	109.0
C10A—C11A—H11C	109.5	C13B—C14B—H14C	109.0
C12A—C11A—H11C	109.5	C15B—C14B—H14D	109.0
H11B—C11A—H11C	108.1	C13B—C14B—H14D	109.0
C13A—C12A—C11A	112.4 (3)	H14C—C14B—H14D	107.8
C13A—C12A—H12A	109.1	C10B—C15B—C14B	111.0 (4)
C11A—C12A—H12A	109.1	C10B—C15B—H15C	109.4
C13A—C12A—H12B	109.1	C14B—C15B—H15C	109.4
C11A—C12A—H12B	109.1	C10B—C15B—H15D	109.4
H12A—C12A—H12B	107.9	C14B—C15B—H15D	109.4
C12A—C13A—C16A	112.0 (3)	H15C—C15B—H15D	108.0
C12A—C13A—C14A	109.7 (3)	C17B—C16B—C13B	116.9 (3)
C16A—C13A—C14A	109.5 (3)	C17B—C16B—H16A	108.1
C12A—C13A—H13B	108.5	C13B—C16B—H16A	108.1
C16A—C13A—H13B	108.5	C17B—C16B—H16D	108.1
C14A—C13A—H13B	108.5	C13B—C16B—H16D	108.1
C15A—C14A—C13A	112.6 (3)	H16A—C16B—H16D	107.3
C15A—C14A—H14A	109.1	C18B—C17B—C16B	111.7 (3)
C13A—C14A—H14A	109.1	C18B—C17B—C22B	110.3 (3)
C15A—C14A—H14B	109.1	C16B—C17B—C22B	110.3 (3)
C13A—C14A—H14B	109.1	C18B—C17B—H17B	108.1
H14A—C14A—H14B	107.8	C16B—C17B—H17B	108.1
C10A—C15A—C14A	110.8 (3)	C22B—C17B—H17B	108.1
C10A—C15A—H15A	109.5	C17B—C18B—C19B	112.8 (3)
C14A—C15A—H15A	109.5	C17B—C18B—H18C	109.0
C10A—C15A—H15B	109.5	C19B—C18B—H18C	109.0
C14A—C15A—H15B	109.5	C17B—C18B—H18D	109.0
H15A—C15A—H15B	108.1	C19B—C18B—H18D	109.0
C13A—C16A—C17A	117.0 (3)	H18C—C18B—H18D	107.8
C13A—C16A—H16B	108.1	C20B—C19B—C18B	111.0 (3)
C17A—C16A—H16B	108.1	C20B—C19B—H19C	109.4
C13A—C16A—H16C	108.1	C18B—C19B—H19C	109.4
C17A—C16A—H16C	108.1	C20B—C19B—H19D	109.4
H16B—C16A—H16C	107.3	C18B—C19B—H19D	109.4
C18A—C17A—C22A	109.1 (3)	H19C—C19B—H19D	108.0
C18A—C17A—C16A	112.7 (3)	N2B—C20B—C19B	109.7 (3)
C22A—C17A—C16A	110.2 (3)	N2B—C20B—C21B	109.5 (3)
C18A—C17A—H17A	108.3	C19B—C20B—C21B	109.0 (3)
C22A—C17A—H17A	108.3	N2B—C20B—H20B	109.5
C16A—C17A—H17A	108.3	C19B—C20B—H20B	109.5
C17A—C18A—C19A	111.4 (3)	C21B—C20B—H20B	109.5
C17A—C18A—H18A	109.3	C22B—C21B—C20B	110.3 (3)
C19A—C18A—H18A	109.3	C22B—C21B—H21C	109.6
C17A—C18A—H18B	109.3	C20B—C21B—H21C	109.6
C19A—C18A—H18B	109.3	C22B—C21B—H21D	109.6
H18A—C18A—H18B	108.0	C20B—C21B—H21D	109.6
C20A—C19A—C18A	112.1 (3)	H21C—C21B—H21D	108.1
C20A—C19A—H19A	109.2	C21B—C22B—C17B	113.4 (3)
C18A—C19A—H19A	109.2	C21B—C22B—H22A	108.9
C20A—C19A—H19B	109.2	C17B—C22B—H22A	108.9
C18A—C19A—H19B	109.2	C21B—C22B—H22B	108.9
H19A—C19A—H19B	107.9	C17B—C22B—H22B	108.9
N2A—C20A—C19A	109.8 (3)	H22A—C22B—H22B	107.7
N2A—C20A—C21A	108.0 (3)	N2B—C23B—C24B	122.0 (4)
C19A—C20A—C21A	109.7 (3)	N2B—C23B—H23A	119.0
N2A—C20A—H20A	109.8	C24B—C23B—H23A	119.0
C19A—C20A—H20A	109.8	C25B—C24B—C23B	122.2 (4)
C21A—C20A—H20A	109.8	C25B—C24B—H24B	118.9
C22A—C21A—C20A	110.8 (3)	C23B—C24B—H24B	118.9
C22A—C21A—H21A	109.5	C24B—C25B—C26B	127.1 (4)
C20A—C21A—H21A	109.5	C24B—C25B—H25B	116.4
C22A—C21A—H21B	109.5	C26B—C25B—H25B	116.4
C20A—C21A—H21B	109.5	C27B—C26B—C31B	117.7 (3)
H21A—C21A—H21B	108.1	C27B—C26B—C25B	122.7 (3)
C17A—C22A—C21A	112.3 (3)	C31B—C26B—C25B	119.5 (4)
C17A—C22A—H22C	109.1	C28B—C27B—C26B	120.9 (4)
C21A—C22A—H22C	109.1	C28B—C27B—H27A	119.5
C17A—C22A—H22D	109.1	C26B—C27B—H27A	119.5
C21A—C22A—H22D	109.1	C27B—C28B—C29B	120.2 (4)
H22C—C22A—H22D	107.9	C27B—C28B—H28B	119.9
N2A—C23A—C24A	121.8 (4)	C29B—C28B—H28B	119.9
N2A—C23A—H23C	119.1	C30B—C29B—C28B	119.8 (4)
C24A—C23A—H23C	119.1	C30B—C29B—H29B	120.1
C25A—C24A—C23A	122.8 (4)	C28B—C29B—H29B	120.1
C25A—C24A—H24A	118.6	C29B—C30B—C31B	120.1 (4)
C23A—C24A—H24A	118.6	C29B—C30B—H30A	119.9
C24A—C25A—C26A	127.5 (4)	C31B—C30B—H30A	119.9
C24A—C25A—H25A	116.2	C30B—C31B—C26B	121.1 (4)
C26A—C25A—H25A	116.2	C30B—C31B—H31B	119.4
C27A—C26A—C31A	117.6 (3)	C26B—C31B—H31B	119.4
			
C6A—C1A—C2A—C3A	0.3 (6)	C6B—C1B—C2B—C3B	0.6 (6)
C1A—C2A—C3A—C4A	−0.2 (6)	C1B—C2B—C3B—C4B	0.8 (7)
C2A—C3A—C4A—C5A	0.8 (6)	C2B—C3B—C4B—C5B	−1.1 (7)
C3A—C4A—C5A—C6A	−1.6 (6)	C3B—C4B—C5B—C6B	0.0 (6)
C4A—C5A—C6A—C1A	1.8 (6)	C2B—C1B—C6B—C5B	−1.7 (6)
C4A—C5A—C6A—C7A	−177.9 (4)	C2B—C1B—C6B—C7B	177.7 (4)
C2A—C1A—C6A—C5A	−1.1 (6)	C4B—C5B—C6B—C1B	1.4 (6)
C2A—C1A—C6A—C7A	178.5 (4)	C4B—C5B—C6B—C7B	−178.0 (4)
C5A—C6A—C7A—C8A	160.1 (4)	C1B—C6B—C7B—C8B	−1.7 (6)
C1A—C6A—C7A—C8A	−19.5 (6)	C5B—C6B—C7B—C8B	177.7 (4)
C6A—C7A—C8A—C9A	−174.6 (4)	C6B—C7B—C8B—C9B	−177.0 (4)
C10A—N1A—C9A—C8A	−175.9 (3)	C10B—N1B—C9B—C8B	−177.5 (4)
C7A—C8A—C9A—N1A	167.4 (4)	C7B—C8B—C9B—N1B	175.9 (4)
C9A—N1A—C10A—C15A	100.8 (4)	C9B—N1B—C10B—C15B	115.4 (4)
C9A—N1A—C10A—C11A	−137.4 (4)	C9B—N1B—C10B—C11B	−123.6 (4)
N1A—C10A—C11A—C12A	−179.2 (3)	N1B—C10B—C11B—C12B	−178.5 (3)
C15A—C10A—C11A—C12A	−57.6 (4)	C15B—C10B—C11B—C12B	−57.2 (5)
C10A—C11A—C12A—C13A	57.2 (5)	C10B—C11B—C12B—C13B	56.3 (5)
C11A—C12A—C13A—C16A	−176.1 (3)	C11B—C12B—C13B—C14B	−53.0 (5)
C11A—C12A—C13A—C14A	−54.2 (4)	C11B—C12B—C13B—C16B	−176.2 (3)
C12A—C13A—C14A—C15A	53.6 (4)	C12B—C13B—C14B—C15B	53.0 (5)
C16A—C13A—C14A—C15A	176.9 (3)	C16B—C13B—C14B—C15B	176.9 (4)
N1A—C10A—C15A—C14A	178.8 (3)	N1B—C10B—C15B—C14B	178.5 (3)
C11A—C10A—C15A—C14A	56.9 (4)	C11B—C10B—C15B—C14B	57.3 (5)
C13A—C14A—C15A—C10A	−55.7 (4)	C13B—C14B—C15B—C10B	−56.3 (5)
C12A—C13A—C16A—C17A	−64.4 (4)	C12B—C13B—C16B—C17B	−69.5 (4)
C14A—C13A—C16A—C17A	173.6 (3)	C14B—C13B—C16B—C17B	167.6 (3)
C13A—C16A—C17A—C18A	−70.6 (5)	C13B—C16B—C17B—C18B	−63.9 (4)
C13A—C16A—C17A—C22A	167.3 (4)	C13B—C16B—C17B—C22B	173.1 (3)
C22A—C17A—C18A—C19A	−55.6 (4)	C16B—C17B—C18B—C19B	−174.0 (3)
C16A—C17A—C18A—C19A	−178.3 (3)	C22B—C17B—C18B—C19B	−50.9 (5)
C17A—C18A—C19A—C20A	56.6 (4)	C17B—C18B—C19B—C20B	56.2 (5)
C23A—N2A—C20A—C19A	−138.2 (3)	C23B—N2B—C20B—C19B	−128.1 (4)
C23A—N2A—C20A—C21A	102.3 (4)	C23B—N2B—C20B—C21B	112.2 (4)
C18A—C19A—C20A—N2A	−173.9 (3)	C18B—C19B—C20B—N2B	−179.1 (3)
C18A—C19A—C20A—C21A	−55.4 (4)	C18B—C19B—C20B—C21B	−59.2 (4)
N2A—C20A—C21A—C22A	175.1 (3)	N2B—C20B—C21B—C22B	179.1 (3)
C19A—C20A—C21A—C22A	55.5 (4)	C19B—C20B—C21B—C22B	59.0 (4)
C18A—C17A—C22A—C21A	57.0 (5)	C20B—C21B—C22B—C17B	−56.4 (5)
C16A—C17A—C22A—C21A	−178.8 (3)	C18B—C17B—C22B—C21B	51.5 (5)
C20A—C21A—C22A—C17A	−57.8 (5)	C16B—C17B—C22B—C21B	175.4 (3)
C20A—N2A—C23A—C24A	−176.4 (3)	C20B—N2B—C23B—C24B	−178.1 (3)
N2A—C23A—C24A—C25A	−178.9 (4)	N2B—C23B—C24B—C25B	177.0 (4)
C23A—C24A—C25A—C26A	−179.0 (3)	C23B—C24B—C25B—C26B	−176.3 (3)
C24A—C25A—C26A—C27A	3.0 (6)	C24B—C25B—C26B—C27B	−7.3 (6)
C24A—C25A—C26A—C31A	−176.8 (4)	C24B—C25B—C26B—C31B	171.8 (4)
C31A—C26A—C27A—C28A	−0.8 (6)	C31B—C26B—C27B—C28B	−1.6 (5)
C25A—C26A—C27A—C28A	179.4 (4)	C25B—C26B—C27B—C28B	177.6 (3)
C26A—C27A—C28A—C29A	0.7 (6)	C26B—C27B—C28B—C29B	0.1 (6)
C27A—C28A—C29A—C30A	−0.1 (7)	C27B—C28B—C29B—C30B	1.1 (6)
C28A—C29A—C30A—C31A	−0.4 (7)	C28B—C29B—C30B—C31B	−0.8 (6)
C29A—C30A—C31A—C26A	0.3 (7)	C29B—C30B—C31B—C26B	−0.8 (6)
C27A—C26A—C31A—C30A	0.3 (6)	C27B—C26B—C31B—C30B	1.9 (5)
C25A—C26A—C31A—C30A	−179.9 (4)	C25B—C26B—C31B—C30B	−177.2 (3)

### Hydrogen-bond geometry (Å, °)

**Table:**

*D*—H···*A*	*D*—H	H···*A*	*D*···*A*	*D*—H···*A*
C1CA—H1CA···N1B^i^	1.00	2.21	3.18 (1)	161
C1CB—H1CB···N1A^i^	1.00	2.20	3.17 (1)	165

Symmetry codes: (i) −*x*, −*y*, −*z*+2.
